# Efficiency of a pneumatic device in controlling cuff pressure of polyurethane-cuffed tracheal tubes: a randomized controlled study

**DOI:** 10.1186/1471-2253-13-50

**Published:** 2013-12-26

**Authors:** Emmanuelle Jaillette, Farid Zerimech, Julien De Jonckheere, Demosthenes Makris, Malika Balduyck, Alain Durocher, Alain Duhamel, Saad Nseir

**Affiliations:** 1 Critical Care Center, R. Salengro Hospital, University Hospital of Lille, Rue E. Laine, 59037 Lille cedex, France; 2 Biochemistry and Molecular Biology Laboratory, Biochemistry Division, Pathology and Biology Center, University Hospital of Lille, 59037 Lille cedex, France; 3 Clinical Investigation Center - Innovative Technologies, INSERM CIC-IT 807, University Hospital of Lille, 152 rue du Dr Alexandre Yersin, 59120 Loos, France; 4 Intensive Care Unit, University Hospital of Larisa, University of Thessaly, Biopolis Street, 41110 Larisa, Greece; 5 Biochemistry and Molecular Biology Laboratory, Faculty of Pharmacy, Lille II University, 1 place de Verdun, 59045 Lille, France; 6 Medical Assessment Laboratory, EA 2694, Nord de France University, 1 place de Verdun, 59045 Lille, France; 7 Epidemiology, Public Health and Quality of Care, Nord-de-France University, Lille, France

**Keywords:** Intubation, Polyurethane, Tracheal cuff, Microaspiration, Tracheal injury, Pneumonia

## Abstract

**Background:**

The primary objective of this study was to determine the efficiency of a pneumatic device in controlling cuff pressure (P_cuff_) in patients intubated with polyurethane-cuffed tracheal tubes. Secondary objectives were to determine the impact of continuous control of P_cuff_, and cuff shape on microaspiration of gastric contents.

**Methods:**

Prospective randomized controlled study. All patients requiring intubation and mechanical ventilation ≥48 h were eligible. The first 32 patients were intubated with tapered polyurethane-cuffed, and the 32 following patients were intubated with cylindrical polyurethane-cuffed tracheal tubes. Patients randomly received 24 h of continuous control of P_cuff_ using a pneumatic device (Nosten®), and 24 h of routine care of P_cuff_ using a manometer. Target P_cuff_ was 25 cmH_2_O. P_cuff_ was continuously recorded, and pepsin was quantitatively measured in all tracheal aspirates during these periods.

**Results:**

The pneumatic device was efficient in controlling P_cuff_ (med [IQ] 26 [24, 28] vs 22 [20, 28] cmH_2_O, during continuous control of P_cuff_ and routine care, respectively; p = 0.017). In addition, percentage of patients with underinflation (31% vs 68%) or overinflation (53% vs 100%) of tracheal cuff, and percentage of time spent with underinflation (0.9 [0, 17] vs 14% [4, 30]) or overinflation (0 [0, 2] vs 32% [9, 54]) were significantly (p < 0.001) reduced during continuous control of P_cuff_ compared with routine care.

No significant difference was found in microaspiration of gastric content between continuous control of P_cuff_ compared with routine care, or between patients intubated with tapered compared with cylindrical polyurethane-cuffed tracheal tubes.

**Conclusion:**

The pneumatic device was efficient in controlling P_cuff_ in critically ill patients intubated with polyurethane-cuffed tracheal tubes.

**Trial registration:**

The Australian New Zealand Clinical Trials Registry (NCT01351259)

## Background

Microaspiration of contaminated oropharyngeal secretions and gastric content frequently occurs in intubated critically ill patients, and plays a major role in the pathogenesis of ventilator-associated pneumonia (VAP) [1]. Aspiration of contaminated secretions is followed by tracheobronchial colonization that might progress into VAP depending on quantity, and virulence of microorganisms, and local and general defense mechanisms [2].

During the last decade, significant progress has been achieved in the field of prevention of microaspiration, and VAP [3,4]. Polyurethane-cuffed tracheal tubes were reported to significantly reduce leakage in *in vitro* studies, and microaspiration in intubated critically ill patients [5-7]. In addition, three clinical studies reported significant reduction in VAP, and nosocomial pneumonia rate in patients intubated with polyurethane-cuffed tracheal tubes compared with those intubated with standard polyvinyl chloride-cuffed tracheal tubes [8-10]. However, limitations of these studies, including the use of subglottic aspiration in the intervention group in one study, clinical definition of nosocomial pneumonia, and before-after design should be taken into account. Recent data coming from *in vitro* studies also suggested a beneficial effect of tapered-shaped tracheal cuff in reducing microaspiration [11-13].

Underinflation, and overinflation of tracheal cuff are major risk factors for microaspiration, VAP, and tracheal injury [14-16]. Despite routine control of cuff pressure (P_cuff_) using a manometer, patients intubated with polyvinyl-chloride or polyurethane-cuffed tracheal tubes spend a large amount of time with underinflation and overinflation of tracheal cuff [6,17]. Continuous control of polyvinyl chloride-cuffed tracheal tubes using a pneumatic device was found to be associated with significantly reduced microaspiration of gastric content, and VAP incidence [18]. However, there are several differences between polyvinyl chloride and polyurethane, including thickness and physicochemical properties. Condensation formation in the pilot external balloon is very frequent in patients intubated with polyurethane-cuffed tracheal tubes. Whether condensation formation or other physicochemical properties of polyurethane could influence the efficiency of a pneumatic device in continuously controlling P_cuff_ is unknown. To our knowledge, no study has evaluated the efficiency of a pneumatic or an electronic device in continuously controlling P_cuff_ in patients intubated with polyurethane-cuffed tracheal tubes. Therefore, we conducted this randomized controlled trial to determine the efficiency of a pneumatic device in controlling P_cuff_ in critically-ill patients intubated with polyurethane-cuffed tracheal tube compared with routine care using a manual manometer. The secondary objectives were to determine the impact of continuous control of P_cuff_, and cuff shape, i.e. tapered versus cylindrical, on microaspiration of gastric content.

## Methods

The local institutional review board of the Lille University Hospital approved this study. The patients provided their written consent before randomization. In unconscious patients who were not able to give consent for inclusion in the study at randomization, relatives (next-of-kin) gave assent on every patient’s behalf, and patients were later given the opportunity to withdraw from the study. The study was registered at clinicaltrial.gov (NCT01351259).

### Study design

This prospective randomized controlled cross-over study was conducted in a single 10-bed medical ICU during a 17-month period. Inclusion criteria were age >18 years, intubation in the ICU, and expected duration of invasive mechanical ventilation ≥48 hours. Exclusion criteria were enrolment in another trial, contraindication for semirecumbent position or for enteral nutrition, and intubation before ICU admission.

Primary objective was to determine the efficiency of a pneumatic device in controlling P_cuff_ in patients intubated with polyurethane-cuffed tracheal tubes compared with routine care using a manual manometer. Secondary objectives were to determine the impact of continuous control of P_cuff_, and cuff shape, i.e. tapered versus cylindrical, on microaspiration of gastric contents.

All patients were intubated with polyurethane-cuffed tracheal tubes. Tracheal tube size was 7.5 and 8 in women and men, respectively. The first 32 patients were intubated with a tapered-cuffed tracheal tube (SealGuard®, Mallinckrodt, Athlone, Ireland), and the 32 following patients were intubated with a cylindrical-cuffed tracheal tube (Microcuff®, Kimberly-Clark, Georgia, USA). Patients were randomly assigned to receive continuous control of P_cuff_ for 24 hours followed by routine care for 24 hours, or routine care for 24 hours followed by continuous control P_cuff_ for 24 hours. The target P_cuff_ was 25 cm H_2_O during the two 24-hour periods. A computer-generated random assignment list in balanced blocks of four was used. Sealed opaque individual envelopes containing treatment assignments were numbered sequentially. All caregivers were blinded to the randomization schedule and the block size. Because of the nature of the intervention, physicians and nurses could not be blinded to the randomization arm. However, engineer who performed the analysis of P_cuff_ recording (JD) and physicians who performed pepsin measurement (FZ, and MB) were blinded to study group assignment.

### Tracheal cuff management

Routine care of tracheal cuff was managed according to an internal procedure adapted from the *Société de Réanimation de Langue Française* recommendations [19]. A manual manometer (Ambu® Cuff Pressure Gauge, Ambu A/S, Ballerup, Denmark) was used to check and adjust P_cuff_ every 8 hours. Continuous control of P_cuff_ was performed using a pneumatic device (Nosten®, Leved, St-Maur, France) [20].

### Outcome measurement

In all patients, P_cuff_ and airway pressure were continuously recorded (Physiotrace®, CHRU, Lille, France) at a digitizing frequency of 100 Hz during the 48 hours following randomization, including 24 hours of continuous control of P_cuff_, and 24 hours of routine care. Nurses were blinded to recording data. Pepsin was quantitatively measured in all tracheal aspirates during the same two 24-hour periods. In order to avoid overlap in pepsin results between the two 24-hour periods, tracheal suctioning was always performed at the end of the first periods of P_cuff_ control. Tracheal aspirates were stored at -20°C. Quantitative pepsin measurement was performed by an ELISA technique [18].

### Study population

Measures aiming at preventing microaspiration were used in all patients including protocolized enteral nutrition, and sedation, minimal positive end expiratory pressure of 5 cm H_2_O, and semirecumbent position. Continuous subglottic suctioning was not utilized. Nurses performed tracheal suctioning every 3 hours or more if clinically indicated, using an open tracheal suction system.

### Definitions

The primary end point was the percentage of patients with underinflation (P_cuff_ <20 cm) H_2_O or overinflation (P_cuff_ >30 cm H_2_O) of tracheal cuff. Secondary outcomes included duration of underinflation and overinflation of P_cuff_, coefficient of variation of P_cuff_, and microaspiration of gastric content. P_cuff_ continuous recording data were used to determine the time spent with normal P_cuff_, underinflation, and overinflation of tracheal cuff. The coefficient of variation of P_cuff_ was calculated as standard deviation/mean P_cuff_ × 100. Microaspiration of gastric content was defined by the presence of pepsin at significant level (>200 ng/mL) in at least one tracheal aspirate.

### Statistical analyses

Based on the incidence of underinflation or overinflation of tracheal cuff in patients intubated with polyurethane-cuffed tracheal tubes and receiving routine care of P_cuff_ in our ICU (70%), we estimated an incidence of underinflation or overinflation in patients intubated with polyurethane-cuffed tracheal tubes and receiving continuous control of P_cuff_ of 30%. Randomly assigning 32 patients to two 24-hour periods of Pcuff control would allow detection of this difference with 80% power and a two-tailed significance level of 0.05. To determine the impact of cuff shape on microaspiration of gastric content, two groups of 32 patients intubated with tapered or cylindrical-cuffed tracheal tubes were required.

All *P* values were two-tailed. Categorical variables were described as frequencies (%). Because they were not normally distributed, continuous variables were described as median (interquartile range). McNemar’s test and Wilcoxon rank test were used to compare qualitative and quantitative variables between the two 24-hour periods, respectively. For comparisons between subgroups (tapered versus cylindrical-cuffed tracheal tubes), χ^2^ test or Fisher exact test were used to compare qualitative variables, and Mann–Whitney *U* test was used to compare continuous variables.

To determine the impact of high airway pressures on the efficiency of the pneumatic device, we compared the time spent with underinflation and overinflation of P_cuff_ during continuous control of tracheal cuff between patients with high airway pressures (≥ 75^th^ quartile of airway pressures in the cohort), and those with lower airway pressures (<75^th^ quartile of airway pressures in the cohort).

## Results

### Patient characteristics

**Figure 1:**
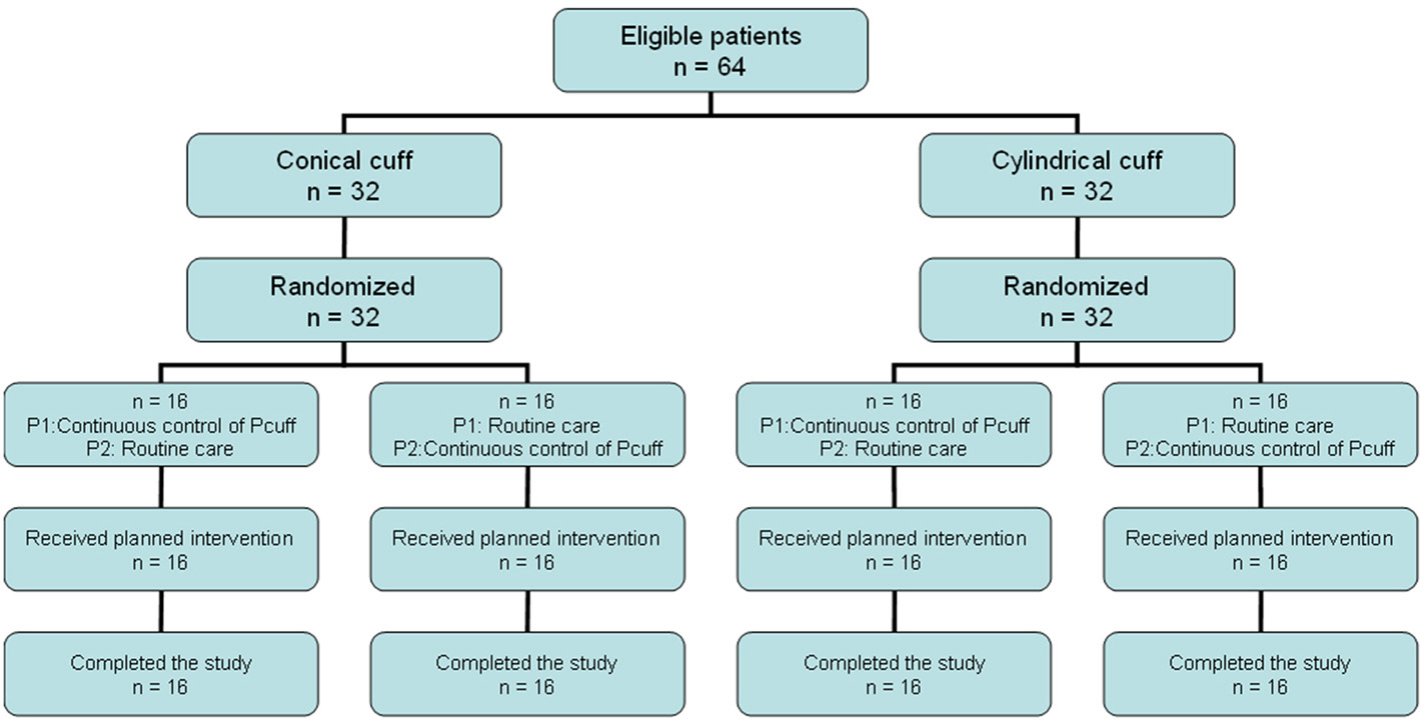
**Study flowchart.** Pcuff, cuff pressure; P1, period 1; P2, period 2.

Sixty four patients were included in this study, including 32 intubated with tapered-cuffed tracheal tube, and 32 with cylindrical-cuffed tracheal tube. Study flow chart is presented in Figure 1. Median time from intubation to randomization was 3 days (1, 7). Median tracheal tube size was 8 mm (7.5-8). No significant difference was found in median time from intubation to randomization (4 [1,8] vs 2 days [1,5]), p = 0.470; or tracheal tube size (8 [7.5-8] vs 8 mm [7.5-8], p = 0.456) between patients intubated with tapered-cuffed tracheal tubes and those intubated with cylindrical-cuffed tracheal tubes, respectively. No significant difference was found in patient characteristics between the two periods of continuous control of P_cuff_ and routine care, or between patients intubated with tapered-cuffed tracheal tubes, and those intubated with cylindrical-cuffed tracheal tubes (Tables 1, and 2).

**Table 1:** Patient characteristics at ICU admission

**Variables**	**All patients n = 64**	**Tapered cuff n = 32**	**Cylindrical cuff n = 32**	P*
Age	66 (55–75)	66 (58–77)	66 (54–72)	0.330
Male gender	43 (67)	20 (62)	23 (71)	0.594
SAPS II	45 (32–62)	45 (29–62)	42 (37–62)	0.289
LOD score	6 (3–9)	6 (2–10)	6 (4–9)	0.818
Comorbidities				
Diabetes mellitus	16 (25)	10 (31)	6 (18)	0.386
COPD	23 (35)	11 (34)	12 (37)	>0.999
Cardiovascular disease	12 (18)	7 (21)	5 (15)	0.749
Cirrhosis	1 (1)	1 (3)	0	>0.999
Causes for ICU admission				
Acute exacerbation of COPD	18 (28)	10 (31)	8 (25)	0.781
ARDS	7 (10)	3 (9)	4 (12)	>0.999
Septic shock	15 (23)	10 (31)	5 (15)	0.238
Community-acquired pneumonia	19 (29)	8 (25)	11 (34)	0.584
Hospital acquired pneumonia	24 (37)	16 (50)	8 (25)	0.071
Congestive heart failure	4 (6)	2 (6)	2 (6)	>0.999
Neurologic failure	7 (10)	2 (6)	5 (15)	0.426
Poisoning	3 (4)	2 (6)	1 (3)	>0.999
Soft tissue infetion	2 (3)	0	2 (6)	0,492

Data are frequencies (%) or median (Interquartile range).

SAPS, simplified acute physiology score; LOD, logistic organ dysfunction; COPD, chronic obstructive pulmonary disease; ARDS, acute respiratory distress syndrome. Some patients had more than one cause for ICU admission.

*P values are for comparisons of tapered with cylindrical cuff.

**Table 2:** Patient characteristics during the 48 hours following randomization

**Variables**	**Continuous control of P**_**cuff **_**n = 64**	**Routine care n = 64**	**p**	**Continuous control of P**_**cuff **_**n = 64**		**p**	**Routine care n = 64**		**p**
				**Tapered cuff n = 32**	**Cylindrical cuff n = 32**		**Tapered cuff n = 32**	**Cylindrical cuff n = 32**	
Quantity of enteral nutrition, ml/day	1000	1000	0.433	1000	1000	0.960	1000	1000	0.241
	(500–1000)	(500–1500)		(500–1000)	(500–1000)		(500–1000)	(500–1500)	
Gastric residual volume, ml	0 (0–20)	0 (0–20)	0.871	0 (0–15)	0 (0–27)	0.726	0 (0–60)	0 (0–5)	0.294
Sedation	28 (43)	29 (45)	> 0.999	14 (43)	14 (43)	>0.999	15 (46)	14 (43)	>0.999
Ramsay score	2 (2–3)	2 (2–3)	0.641	2 (2–3)	2 (2–3)	0.718	2 (2–3)	2 (2–3)	0.729
Neuromuscular blocking agent use	6 (9)	7 (10)	> 0.999	3 (9)	3 (9)	>0.999	3 (9)	4 (12)	>0.999
Glasgow coma score	14 (10–15)	15 (10–15)	0.310	13 (10–15)	14 (8–15)	0.738	14 (10–15)	15 (8–15)	0.908
PEEP	6 (5–8)	6 (5–9)	0.943	6 (5–8)	7 (5–9)	0.234	6 (5–9)	7 (5–9)	0.166
Ventilatory mode			0.329			0.885			0.433
ACV	36 (56)	37 (57)		18 (56)	18 (56)		21 (65)	16 (50)	
PSV	23 (35)	25 (39)		11 (34)	12 (37)		10 (31)	15 (46)	
BPPV	5 (7)	2 (3)		3 (9)	2 (6)		1 (3)	1 (3)	
Stress ulcer prophylaxis or treatment			>0.999			0.688			0.109
Sucralfate	32 (50)	32 (50)		15 (46)	17 (53)		13 (40)	19 (59)	
Proton pump inhibitors	25 (39)	25 (39)		13 (40)	12 (37)		13 (40)	12 (37)	
Prokinetic drugs	12 (18)	12 (18)	> 0.999	6 (18)	6 (18)	>0.999	7 (21)	5 (15)	0.749
Vomiting	3 (4)	1 (1)	0.625	0	3 (9)	0.237	1 (3)	0	>0.999
Head-of-bed position	40 (35–40)	40 (35–40)	0.846	40 (35–45)	40 (35–40)	0.602	40 (35–40)	40 (35–45)	0.244

Data are frequencies (%) or median (Interquartile range).

P_cuff_, cuff pressure; PEEP, positive end-expiratory pressure; ACV, assist-control ventilation; PSV, pressure support ventilation; BPPV, bilevel positive pressure ventilation.

### Continuous control versus routine care of tracheal cuff

The pneumatic device was efficient in controlling P_cuff_. Percentage of patients with underinflation or overinflation of tracheal cuff, coefficient of variation of P_cuff_, and percentage of time spent with underinflation and overinflation of tracheal cuff were significantly lower during continuous control of P_cuff_ compared with routine care. P_cuff_, and percentage of time spent with P_cuff_ 20–30 cmH_2_O were significantly higher during continuous control of P_cuff_ compared with routine control. No significant difference was found in rate of microaspiration of gastric content, mean pepsin level, or percentage of tracheal aspirates positive for pepsin between continuous control of P_cuff_ and routine care (Table 3). No significant interaction was found between continuous control of P_cuff_ and cuff shape (p = 0.93).

**Table 3:** Impact of the pneumatic device on tracheal cuff pressure and microaspiration of gastric content

**Variables**	**Continuous control of P**_**cuff **_**n = 64**	**Routine care n = 64**	**p**	**OR (95% CI)**
Recording duration, h	24 (23–24)	24 (23–24)	0.198	
Mean airway pressure, cm H_2_O	13 (11–18)	13 (11–17)	0.216	
Mean P_cuff_, cm H_2_O	26 (24–28)	22 (20–28)	0.017	
Coefficient of P_cuff_ variation, %	5 (3–12)	19 (15–30)	<0.001	
P_cuff_ 20-30 cm H_2_O				
yes	64 (100)	64 (100)	NA	
% of recording time	95 (70–99)	44 (30–59)	<0.001	
P_cuff_ <20 or >30, cm H_2_O				
Yes	41 (66)	64 (100)	<0.001	
Duration >30 min	33 (51)	64 (100)	<0.001	
P_cuff_ <20, cm H_2_O				
Yes	19 (29)	60 (93)	<0.001	1.46 (1.23-1.73)
Duration > 30 min	16 (25)	57 (89)	<0.001	2.63 (1.11-6.21)
% of recording time	0.01 (0–2)	32 (9–54)	<0.001	
P_cuff_ >30, cm H_2_O				
Yes	33 (51)	62 (96)	0.001	1.57 (1.10-2.24)
Duration >30 min	25 (39)	54 (84)	<0.001	1.50 (1.11-2.20
% of recording time	0.9 (0–17)	14 (4–30)	<0.001	
Microaspiration of gastric content	32 (50)	38 (59)	0.238	
Pepsin, ng/mL	185 (113–296)	203 (120–338)	0.171	
% of tracheal aspirates positive for pepsin	29 (0–74)	45 (0–100)	0.162	

Data are frequencies (%) or median (Interquartile range). P_cuff_, cuff pressure.

Yes indicates that a patient had the variable at least once.

### Tapered versus cylindrical cuff shape during continuous control of P_cuff_

During continuous control of P_cuff_, percentage of patients with underinflation of tracheal cuff >30 minutes was significantly lower in patients intubated with tapered-cuffed tracheal tubes compared with those intubated with cylindrical-cuffed tracheal tubes. No significant difference was found in terms of P_cuff_, percentage of patients with underinflation or overinflation, coefficient of variation of P_cuff_, and percentage of time spent with underinflation or overinflation between the two groups during the same period (Table 4).

**Table 4:** Impact of cuff shape on cuff pressure and microaspiration during continuous control of cuff pressure and routine care

**Variables**	**Continuous control of P**_**cuff **_**n = 64**		**p**	**Routine care n = 64**		**p**
	**Tapered cuff n = 32**	**Cylindrical cuff n = 32**		**Tapered cuff n = 32**	**Cylindrical cuff n = 32**	
Recording duration, h	24 (23–24)	24 (23–25)	0.662	24 (23–25)	24 (23–24)	0.229
Mean airway pressure, cm H_2_O	13 (10–18)	13 (11–16)	0.749	12 (10–20)	15 (11–15)	0.214
Mean P_cuff_, cm H_2_O	26 (25–28)	25 (23–28)	0.844	21 (19–26)	24 (21–30)	0.143
Coefficient of P_cuff_ variation, %	5 (3–9)	6 (3–15)	0.382	18 (15–30)	20 (14–39)	0.789
P_cuff_ 20-30 cm H_2_O						
yes	32 (100)	32 (100)	NA	32 (100)	32 (100)	NA
% of recording time	97 (82–99)	88 (56–99)	0.147	42 (30–62)	44 (29–57)	0.811
P_cuff_ <20 or >30 cm H_2_O						
Yes	19 (59)	22 (68)	0.856	32 (100)	32 (100)	>0.999
Duration >30 min	14 (43)	19 (59)	0.455	30 (93)	32 (100)	>0.999
P_cuff_ <20 cm H_2_O						
Yes	6 (18)	13 (40)	0.116	29 (90)	31 (96)	>0.999
Duration > 30 min	3 (9)	13 (40)	0.011*	29 (90)	28 (87)	0.371
% of recording time	0 (0–0)	0 (0–12)	0.549	41 (17–62)	21 (8–47)	0.049
P_cuff_ >30 cm H_2_O						
Yes	15 (46)	18 (56)	0.903	31 (96)	31 (96)	>0.999
Duration >30 min	12 (37)	13 (40)	>0.999	24 (75)	30 (93)	0.235
% of recording time	0.6 (0–24)	1.4 (0–48)	0.652	11 (2–17)	20 (6–18)	0.031
Microaspiration of gastric content	13 (40)	19 (59)	0.211	18 (56)	20 (62)	0.799
Pepsin, ng/mL	154 (105–230)	214 (126–466)	0.066	175 (121–252)	238 (101–664)	0.238
% of tracheal aspirates positive for pepsin	0 (0–57)	43 (0–100)	0.073	40 (0–86)	50 (0–100)	0.310

Data are frequencies (%) or median (Interquartile range).

P_cuff_, cuff pressure. Yes indicates that a patient had the variable at least once.

*OR (95% CI) 3.20 (1.12-9.12).

### Tapered versus cylindrical cuff shape during routine care of P_cuff_

During routine care, percentage of time spent with underinflation of tracheal cuff was significantly higher, and percentage of time spent with overinflation of tracheal cuff was significantly lower in patients intubated with tapered-cuffed tracheal tubes compared with those intubated with cylindrical-cuffed tracheal tubes. No significant difference was found in coefficient of variation of P_cuff_, or percentage of patients with underinflation or overinflation of tracheal cuff between the two groups during the same period (Table 4).

### Impact of cuff shape on microaspiration

No significant difference was found in microaspiration of gastric content rate, mean pepsin level or percentage of tracheal aspirates positive for pepsin between patients intubated with tapered-cuffed tracheal tubes and those intubated with cylindrical-cuffed tracheal tubes during continuous control of P_cuff_ or routine care (Table 4).

### Other results

During continuous control of P_cuff_, important condensation was observed in the pilot balloon in 7 (22%) patients intubated with tapered-cuffed tracheal tube compared with 10 (31%) patients intubated with cylindrical-cuffed tracheal tube (p = 0.571).

No significant difference was found in percentage of time spent with underinflation (median [IQR] 0.07% [0.0004-16] versus 0.01% [0.0003-0.6], p = 0.359), or overinflation of tracheal cuff (6% [0.27-37] versus 0.34% [0.02-11]) between patients with high airway pressures (n = 16) and those with lower airway pressures (n = 48), respectively.

## Discussion

The percentage of patients with underinflation or overinflation of tracheal cuff, percentage of time spent with underinflation or overinflation of tracheal cuff, and coefficient of variation of P_cuff_ were significantly lower during continuous control of P_cuff_ compared with routine care. Further, percentage of time spent with P_cuff_ 20–30 cmH_2_O, and mean P_cuff_ were significantly higher during continuous control of P_cuff_ compared with routine care. However, no significant difference was found in the incidence of microaspiration of gastric content between continuous control of P_cuff_ compared with routine care or between patients intubated with tapered-cuffed tracheal tubes and those intubated with cylindrical-cuffed tracheal tubes.

To our knowledge, our study is the first to demonstrate the efficiency of the pneumatic device in controlling P_cuff_ in critically-ill patients intubated with polyurethane-cuffed tracheal tubes. Previous studies found similar results in animals and humans intubated with polyvinyl chloride-cuffed tracheal tubes [20,21]. The percentage of patients with underinflation of tracheal cuff >30 minutes during continuous control of P_cuff_ was relatively high (25%). This could be explained by the physicochemical characteristics of polyurethane, namely its hydrophilic aspect, resulting in condensation formation especially during continuous control of P_cuff_. In our study, small condensation formation was rarely observed in the pilot balloon during routine care of P_cuff_. However, important condensation formation filling the whole pilot balloon was frequently observed during continuous control of P_cuff_. The fact that evaporation is probably reduced in closed circuit during continuous control of P_cuff_ might explain this difference in condensation formation between routine care and continuous control of P_cuff_. Further studies directly comparing efficiency of the pneumatic device in polyurethane and polyvinyl chloride-cuffed tracheal tubes are needed to confirm our results. Although previous studies have demonstrated the efficiency of the pneumatic device in controlling P_cuff_ in patients intubated with PVC-cuffed tracheal tubes, the current study allow the generalization of this observation to patients intubated with polyurethane-cuffed tracheal tubes. Given the growing evidence supporting the use of polyurethane-cuffed tracheal tubes to prevent microaspiration and VAP, our results are necessary before performing further studies aiming to evaluate the combined beneficial effects of using polyurethane-cuffed tracheal tubes and continuous control of P_cuff_ in preventing VAP.

In spite of efficient control of P_cuff_, no significant difference was found in microaspiration of gastric content between continuous control of P_cuff_ and routine care. One potential explanation for this result is the optimal routine care provided during the study, and the use of polyurethane-cuffed tracheal tubes. These tubes have been demonstrated to significantly reduce microaspiration in critically ill patients [5,6]. Further, our study is probably underpowered to detect such an effect, since microaspiration of gastric content was a secondary outcome and the number of included patients was calculated based on the primary outcome. Further studies are needed to determine the impact of continuous control of P_cuff_ on the incidence of microaspiration, and VAP. However, whilst our study was underpowered to detect a difference in microaspiration, it was correctly powered to draw a valuable conclusion on primary outcome, i.e. the efficiency of the pneumatic device in controlling P_cuff_. In fact, the number of patients required to detect the estimated difference in percentage of patients with underinflation or overinflation of P_cuff_, with an alpha risk of 5% and a power of 80%, was 32 per group. As 32 patients were included in each study group, our study is sufficiently powered to conclude that the pneumatic device is efficient in controlling P_cuff_. This result would allow future studies to evaluate the impact of the pneumatic device on the prevention of complications related to underinflation and overinflation of P_cuff_. Although our previous study has already suggested beneficial effects of continuous control of P_cuff_ in reducing microaspiration and VAP [18], it was conducted in patients intubated with PVC-cuffed tracheal tubes. Therefore, these results could not be generalized to patients intubated with polyurethane-cuffed tracheal tubes. Further studies with larger sample size are required in these patients to determine the impact of continuous control of P_cuff_ on prevention of cuff-related complications such as microaspiration, and VAP.

Some significant differences, including percentage of time with underinflation and overinflation were found between patients intubated with tapered-cuffed tracheal tubes compared with those intubated with cylindrical-cuffed tracheal tubes. These differences could be explained by different cuff shape between the two groups. No significant effect was found of tracheal cuff shape on microaspiration of gastric content. However, during continuous control of P_cuff_ a trend towards lower pepsin level and percentage of tracheal aspirates positive for pepsin was found in patients intubated with tapered-cuffed tracheal tubes compared with those intubated with cylindrical-cuffed tracheal tubes. Further, in spite of significantly higher percentage of time spent with underinflation of tracheal cuff in patients intubated with tapered-cuffed tracheal tubes compared with those intubated with cylindrical-cuffed tracheal tubes during routine care, microaspiration of gastric content rate was similar in the two groups. This result also suggests better sealing with tapered compared with cylindrical tracheal cuffs. However, our study was probably underpowered to detect a significant difference in microaspiration rate between the two groups.

Two recent *in vitro* studies found tapered-shaped tube cuff to considerably improve sealing characteristics of polyvinyl chloride tube cuffs [11,13]. However, no significant effect of the tapered-shaped cuff was found in polyurethane tube cuffs. In contrast, another *in vitro* study reported that tapered polyurethane cuff was more efficient than cylindrical polyurethane cuff in larger tracheal diameter in preventing fluid leakage [12]. Different study design might explain these conflicting results. In a prospective observational before-after study, our group found no significant difference in microaspiration of gastric content between patients intubated with tapered polyurethane-cuffed tracheal tubes compared with those intubated with cylindrical polyurethane-cuffed tracheal tubes [6]. Further clinical studies are required to determine the impact of tracheal cuff shape on the incidence of microaspiration, and VAP.

No significant difference was found in airway pressures between patients intubated with tapered compared with those intubated with cylindrical tracheal cuffs. Previous studies demonstrated that P_cuff_ was tightly correlated to airway pressure [22,23]. The relatively low airway pressures found in study patients were similar as those previously reported in other ICU patients [6,24,25]. This might strengthen our findings, since these results could be generalized to patients with similar airway pressure. Percentage of time spent with underinflation, and overinflation of P_cuff_ during continuous control of P_cuff_ was not significantly different between patients with high airway pressures and those with lower airway pressures, suggesting that the pneumatic device is efficient in patients with high airway pressures, such as those with severe asthma or ARDS. However, no definite conclusion could be drawn in this subgroup because of the small number of patients (n = 16).

Our study has some limitations. First, this study was performed in a single ICU. Therefore, our results could not be generalized to other ICU patients. Second, our study did not evaluate the impact of continuous control of P_cuff_ on outcomes such as VAP or tracheal injury. However, in order to adjust for patient-related confounders, such as tracheal size and aspect, airway pressures, and tracheal tube size, continuous control and routine care of P_cuff_ were performed in the same patient. Therefore, it was not possible to evaluate these outcomes. Further, it was mandatory to evaluate the efficiency of the device in controlling P_cuff_ before performing studies on its impact on prevention of complications. Finally, randomization for tracheal cuff shape was performed per period and not per patient. However, intubation is often an urgent procedure. Therefore, it is very difficult to perform randomization per patient in such a study. In addition, the impact of cuff shape on microaspiration was a secondary outcome.

## Conclusion

We conclude that the pneumatic device is efficient in controlling P_cuff_ in critically ill patients intubated with polyurethane-cuffed tracheal tube. Further studies are needed to determine the impact of continuous control of P_cuff_ and tracheal cuff shape on microaspiration, VAP, and tracheal injury.

### Key messages

• The pneumatic device is efficient in controlling P_cuff_ in critically ill patients intubated with polyurethane-cuffed tracheal tube.

• Conical cuff shape could be beneficial in preventing microaspiration in critically ill patients.

• Further studies are needed to determine the impact of continuous control of P_cuff_ and tracheal cuff shape on microaspiration, and VAP in patients intubated with polyurethane-cuffed tracheal tubes.

## Abbreviations

ICU: Intensive care unit; Pcuff: Cuff pressure; VAP: Ventilator-associated pneumonia.

## Competing interests

SN: Covidien (advisory board), other authors: none.

## Authors’ contribution

EJ, ADur, and SN designed this study. EJ, FZ, JD, MB, and SN collected the data. ADuh performed statistical analyses. EJ, and SN wrote the manuscript, and all authors participated in its critical revision. EJ, and SN had full access to all data in the study and had final responsibility for the decision to submit for publication. All authors read and approved the final manuscript.

## Pre-publication history

The pre-publication history for this paper can be accessed here:

http://www.biomedcentral.com/1471-2253/13/50/prepub
